# Effect of Efflux Pump Inhibitor Carbonyl Cyanide 3-Chlorophenylhydrazone on the Minimum Inhibitory Concentration of Ciprofloxacin in *Acinetobacter baumannii* Clinical Isolates

**DOI:** 10.5812/jjm.8691

**Published:** 2014-01-01

**Authors:** Abdollah Ardebili, Malihe Talebi, Leila Azimi, Abdolaziz Rastegar Lari

**Affiliations:** 1 Department of Microbiology, Faculty of Medicine, Iran University of Medical Sciences, Tehran, IR Iran; 2 Antimicrobial Resistance Research Center, Iran University of Medical Sciences, Tehran, IR Iran

**Keywords:** *Acinetobacter baumannii*, Burn, Ciprofloxacin, Efflux Pumps, Carbonyl cyanide 3-chlorophenylhydrazone

## Abstract

**Background:**

*Acinetobacter baumannii* is an important human pathogen with increasing notoriety in the recent years, as a causative organism of drug resistant nosocomial infections, particularly in immunocompromised patients hospitalized in burn centers.

**Objectives:**

The aim of this study was to determinate the role of efflux pump(s) in ciprofloxacin resistance of *A. baumannii* strains isolated from burn patients.

**Materials and Methods:**

Sixty-five *A. baumannii* strains were isolated from the burn patients hospitalized in Motahari Burns and Reconstruction Center in Tehran, Iran. Susceptibility test to ciprofloxacin was carried out by disk agar diffusion and agar dilution methods, according to the CLSI guidelines. Activity of the efflux system was evaluated using efflux pump inhibitor carbonyl cyanide 3-chlorophenylhydrazone (CCCP).

**Results:**

All *Acinetobacter* isolates were resistant to ciprofloxacin. The Minimum inhibitory concentration (MIC) range of ciprofloxacin in isolates was 4 to 128 µg/mL or greater. Moreover, susceptibility of strains to ciprofloxacin was highly increased in the presence of efflux pump inhibitor; So that, for 86.1% (56/65) of isolates, CCCP reduced the MIC by 2 to 64 folds.

**Conclusions:**

Our findings are suggestive that efflux-based system may play a role in fluoroquinolone resistance in *A. baumannii* isolates, affecting hospitalized patients. The ability of *Acinetobacter* to acquire resistance to these potent antimicrobials by the efflux pump mechanism is a concern. Therefore, new strategies are required in order to eliminate the efflux transport activity from the resistant bacteria causing nosocomial infections and provide more appropriate approaches for treatment and management of troubling infections.

## 1. Background

Over the past three decades, *Acinetobacter baumannii* has emerged as a serious nosocomial pathogen, especially in hospitalized burned patients worldwide (1). Today, this bacterium and *Pseudomonas aeruginosa* are the predominant isolated organisms of burn patients in many countries, including Iran (2). Certain strains of *A. baumannii* are now resistant to many commonly-used antibiotics, including fluoroquinolones, and multidrug resistance is often responsible for the failure of antibiotic therapy (3, 4). Resistance to fluoroquinolones is mediated mainly by chromosomal mutations in gyrA and parC genes that are associated with high levels of resistance (5, 6). Another mechanism responsible for fluoroquinolones resistance is reduction in the drug accumulation due to either impermeability of outer membrane or overexpression of active efflux pumps (1, 7-10).

Efflux systems are composed of transport proteins that pump out a broad range of toxic substrates such as antibiotics and biocides from bacteria, in an energy-dependent manner (6). In these circumstances, the intracellular antibiotic concentration is decreased and bacteria become less susceptible to that compound. The minimum inhibitory concentrations (MICs) of antibiotics for strains overexpressing an efflux pump, are usually 2 to 8 folds higher than those for susceptible strains of that species (10, 11).

To assess the role of drug efflux mechanism in bacteria, efflux pump inhibitors (EPIs) are widely used to totally abolish the efflux of various molecules (10, 12). One of these compounds is carbonyl cyanide 3-chlorophenylhydrazone (CCCP) (an uncoupler of oxidative phosphorylation which disrupts the proton gradient of the membranes) that has often been found to increase the susceptibility of a number of multidrug resistant bacteria, including *A. baumannii* (9, 13, 14).

## 2. Objectives

There are several documented studies on fluoroquinolones resistance in clinical isolates of *A. baumannii* from Iranian populations, but the role of *A. baumannii* efflux pump in resistance to fluoroquinolones has not been investigated. So, the aim of this study was to analyze the contribution of active efflux system to ciprofloxacin resistance in clinical isolates of *A. baumannii* using the efflux pump inhibitor CCCP.

## 3. Materials and Methods

### 3.1. Patients and Bacterial Isolates

**Figure 1.**
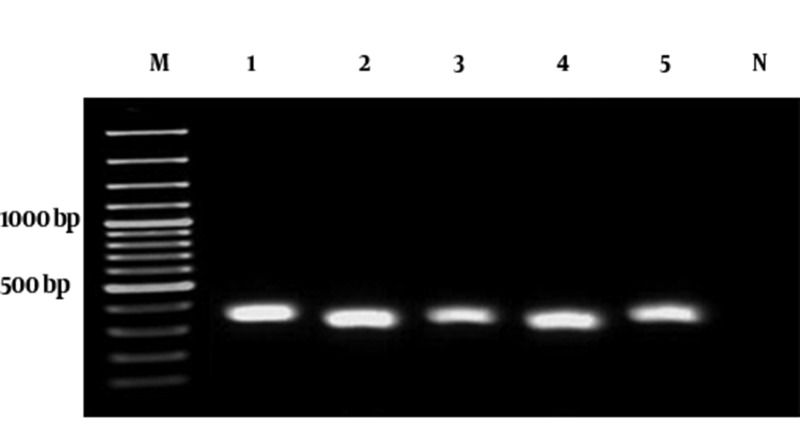
PCR Amplification of *blaOXA-51-Like *Gene in Five Selected Isolates of *A. baumannii * Lanes: M, 100 bp DNA Ladder; 1 - 5, PCR products of *blaOXA-51-like* gene (353 bp); N, negative control (*A. lwoffii*).

In a six-month study, a total of 164 burn patients, hospitalized at least 2 weeks at Motahari Burns and Reconstruction Center in Tehran, were participated. The age range of the patients was between 1 and 88 years, the burn degree was at least II, and in most of them, the total body surface area (TBSA) was more than 10%. After sampling the burn wounds, clinical specimens were examined microbiologically. Bacterial isolates were identified as *A. baumannii *based on the standard biochemical tests following the criteria of Bouvet and Grimont (15) and then confirmed by PCR amplification of *blaOXA-51-like *gene (Figure 1) (16 - 18). 

### 3.2. PCR Amplification of blaOXA-51-Like Gene

PCR analysis of blaOXA-51-like carbapenemase gene was used in order to confirm the presence of *A. baumannii* species (16). Extraction of genomic DNA from bacterial isolates was done by the genomic DNA purification kit (Fermentas, Germany) according to the manufacturer's instructions. The primer pair, 5'-TAATGCTTTGATCGGCCTTG-3' and 5'-TGGATTGCACTTCATCTTGG-3' was used for gene amplification. The PCR conditions were as follows: initial denaturation at 94ºC for 5 minutes; 30 cycles with denaturation at 94ºC for 45 seconds, annealing at 58ºC for 1 minute, and extension at 72ºC for 1 minute, followed by final extension at 72ºC for 5 minutes. The PCR products were detected by electrophoresis in agarose gel (2, w/v %) containing 0.5 µg/mL of ethidium bromide.

### 3.3. Antimicrobial Susceptibility Testing

Susceptibility of the *A. baumannii* isolates to ciprofloxacin (5 µg) (Mast, Merseyside, UK) was initially tested using the standard disk agar diffusion method on Mueller-Hinton (M-H) agar plates. Then, the MIC of ciprofloxacin against bacterial isolates was evaluated by the agar dilution technique. Both of these methods were carried out according to the guidelines established by the clinical and laboratory standards institute (CLSI) (19). The standard strain of *A. baumannii* 19606 was used for quality control of the susceptibility studies.

### 3.4. Treatment of the Efflux Pump Inhibitor

To explore the presence of efflux pump mechanism, efflux pump inhibitor CCCP was added to each of M-H agar plates containing 0.5 to 128 µg/mL ciprofloxacin. The final concentration of CCCP in the M-H agar was 25 µg/mL (20). Then, MIC for ciprofloxacin was determined again. A plate containing CCCP and not containing antibiotics was used as control. The positive criterion for the presence of efflux pump in isolates was decrease of at least 4 folds of ciprofloxacin MIC after the CCCP addition (20).

## 4. Results

### 4.1. Bacterial Isolates and Ciprofloxacin Resistance

In a six months period, 65 strains of *A. baumannii *were isolated from hospitalized burn patients using biochemical tests and confirmatory PCR assays. The PCR-amplified DNA products of blaOXA-51-like carbapenemase gene from five selected clinical isolates are shown in Figure 1. The preliminary results of ciprofloxacin susceptibility test using the disk agar diffusion method showed that all of the *A. baumannii *strains were resistant to this antibiotic. The MIC for ciprofloxacin in bacterial isolates is shown in Table 1. According to the established breakpoint values recommended by CLSI (19), the *A. baumannii *isolates with MIC ≥ 4 µg/mL are considered as ciprofloxacin resistant. In the present study, *Acinetobacter *isolates had a ciprofloxacin MIC range between 4 to 128 µg/mL or greater; so that, the MICs for 6.1% (4/65), 43% (28/65) and 10.7% (7/65) of isolates were 4, 128 and >128 µg/mL, respectively. 

**Table 1.** Effects of CCCP on the Ciprofloxacin MIC in *A. baumannii *Isolates

Isolates, No. (%)	MIC ^a^-CCCP ^a^, µg/mL	MIC Ranges, µg/mL + CCCP	Fold Reduction in MIC + CCCP
**4 (6.1)**	4	1 - 2	2 - 4
**3 (4.6)**	8	1 - 4	2 - 8
**1 (1.5)**	16	8	2
**7 (10.7)**	32	4 - 32	0 - 8
**15 (23)**	64	1 - 64	0 - 64
**28 (43)**	128	32 - 128	0 - 4
**7 (10.7)**	> 128	32 - 128	At least 2

^a^ Abbreviations: MIC, Minimum inhibitory concentration; CCCP, Carbonyl cyanide 3-chlorophenylhydrazone.

### 4.2. Effects of the Efflux Pump Inhibitor on Ciprofloxacin Resistance

To determinate the role of efflux pump in the ciprofloxacin resistant phenotypes in 65 *A. baumannii *isolates, we evaluated the MIC of ciprofloxacin in the presence of 25 µg/mL CCCP, and then, compared the MICs with and without CCCP. Results indicated that most of the isolates (86.1%) became less resistant (2 to 64 folds) to ciprofloxacin in the presence of efflux pump inhibitor (Table 1). Based on a 4-fold or greater decrease in the MIC as the criterion for significance (15), the MIC for 30 of the 65 isolates (46.1%) was reduced significantly 4 to 64 folds. In addition, when the efflux pump inhibitor was added, 26 isolates (40%) exhibited a 2-fold MIC change, while 9 (13.8%) did not change. All bacteria grew well in the M-H agar plates with CCCP that did not contain ciprofloxacin, indicating that 25 µg/mL CCCP did not have an antibacterial effect itself. These results showed that the drug efflux systems are associated with resistance to ciprofloxacin in *A. baumannii *isolates. 

## 5. Discussion

*Acinetobacter* species, especially *A. baumannii*, are responsible for hospital-acquired infections. Nowadays, antimicrobial resistance in this bacterium has become an important issue worldwide. Resistance rates to most antibiotics such as fluoroquinolones, as one of the first-line drugs to treat *A. baumannii* infections, are increasing globally, including in Iran (21-24). In a survey conducted by Wang and colleagues in Taiwan, all *A. baumannii* isolates were resistant to ciprofloxacin and other antibiotics (21). Additionally, in a six-year prospective study in Iran, it was found that only 20.1% of *A. baumannii* isolates were susceptible to ciprofloxacin and susceptibility rate to this antibiotic reduced gradually among *Acinetobacter* isolates in Iran (22).

Asadollahi et al. during 2009 and 2010 determined that 100% of *A. baumannii* isolates were resistant to ciprofloxacin (25). Similar to the mentioned studies, all of our isolates showed resistance to ciprofloxacin. However, resistance rate observed in the present investigation was lower than that of UK and China (50.9% and 61.2%, respectively) (4, 26). This discrepancy could be due to differences in the quality programs of antimicrobial susceptibility, patterns of antibiotic usage, geographic conditions, and environmental factors in various countries. Furthermore, given the MIC values in our study, similar to those reported by Valentine et al. (3), emergence of high-level resistant *A. baumannii* strains to ciprofloxacin among the hospitalized burn patients in Tehran is of concerns.

There are increasing evidences that drug efflux pumps are a mechanism of resistance in a number of clinically-important bacteria, including *A. baumannii* (7-11). Although high level resistance may not occur as a result of multidrug efflux pumps alone, the overexpression association of these genes among highly-resistant clinical isolates cannot be ignored. Efflux pump inhibitors have been shown to reverse multidrug resistance in *A. baumannii* and other bacteria (10). The effects of these compounds, such as CCCP, on the antimicrobial susceptibility were examined in some studies (1, 27). Rajamohan and coworkers found that addition of CCCP at final concentration of 25 µg/mL greatly reduced the MIC of various biocides from 2 to 12 folds (27). In consistence with the results obtained by Lin et al. (9), we observed that the ciprofloxacin susceptibility of most isolates was increased in the presence of CCCP, mainly 2 to 4 folds.

These results suggest that drug efflux pumps are involve in resistance to fluoroquinolone in clinical isolates of *A. baumannii*. AdeABC is an efflux pump described in *A. baumannii,* overexpression of which confers resistance to fluoroquinolones and other antimicrobial agents. Further studies are necessary to clarify the role of this efflux pump overexpression on fluoroquinolone resistance in *A. baumannii* (8-10). In this regard, detection of the AdeABC efflux pump by PCR and determination of the role of its alterations in fluoroquinolone resistance of *Acinetobacter* strains will be a part of our researches in the future.

In conclusion, it seems from the present and other local studies that the prevalence of fluoroquinolone-resistant *A. baumannii* strains has recently increased in Iranian hospitals. Furthermore, results of our investigation are suggestive that drug efflux system has a role in conferring resistance to fluoroquinolone in *Acinetobacter* isolates and this mechanism is getting widespread in clinical settings as well as among hospitalized patients, especially in burn units. So, efforts should be aimed at detecting such resistant bacteria along with their resistance mechanisms, controlling infections caused by them, and finally, providing better alternative therapies against these recalcitrant organisms.
